# From Olive Pomace to Squalane: A Green Chemistry Route Using 2‑Methyltetrahydrofuran

**DOI:** 10.1021/acsomega.5c03685

**Published:** 2025-07-25

**Authors:** Christian Cravotto, Fabio Bucciol, Jean-Baptiste Mazzitelli, Emmanuel Petitcolas, Anne-Sylvie Fabiano-Tixier, Silvia Tabasso

**Affiliations:** † Department of Drug Science and Technology, 9314 University of Turin, Via P. Giuria 9, 10125 Turin, Italy; ‡ Avignon Université, INRAE, UMR SQPOV, F-84000 Avignon, France

## Abstract

Squalene (SQE) is a key triterpene used in pharmaceuticals, nutraceuticals and cosmetics. Although olive pomace (OP) is a sustainable source of SQE, conventional hexane extraction raises environmental and health concerns. This study investigates the potential of 2-methyltetrahydrofuran (2-MeTHF) as a greener alternative for SQE extraction and catalytic hydrogenation to squalane (SQA); a high-value compound in industrial applications. 2-MeTHF provided 83% SQE recovery from OP, which was further concentrated in deodorizer distillates during refining. SQE hydrogenation in 2-MeTHF significantly improved reaction efficiency at lower temperatures (60 °C, 3 bar H_2_, 0.5 mol % Pd/C), enabling full conversion within 1 h. This represents a major advantage over conventional industrial hydrogenation, which requires harsher conditions (200 °C, 4–30 bar H_2_) and longer reaction times (6–7 h). In order to assess industrial feasibility, SQE from OP deodorizer distillates (6.8 wt %) was concentrated via saponification and molecular distillation (∼34 wt %), followed by flash chromatography (59 wt % purity, 85% recovery). However, residual impurities caused catalyst poisoning, lowering the SQA yield to 19.8%. This study highlights 2-MeTHF’s potential for industrial-scale SQE valorization via integrated extraction and hydrogenation. Future efforts should focus on improving SQE purification from OP-DDs and enhancing catalyst recyclability.

## Graphical abstract

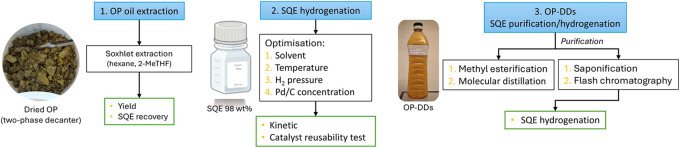

## 1 Introduction

Squalene (SQE) is a linear triterpene that is frequently used in pharmaceuticals, food supplements and cosmetics due to its multiple functions.^1^ SQE acts as a biochemical precursor to sterols and terpenoids, which play a central role in the functions of plants, animals and humans.^2^

Originally identified in the early 20th century by Tsujimoto in deep-sea shark liver oils, SQE is known for its anticancer, antioxidant, detoxifier and skin emollient activities.^3^ SQE has traditionally been extracted from shark liver oil (40–80% of the total oil), raising conservation concerns due to declining shark populations.^4^ SQE of over 98 wt % purity is obtained from shark oil through a single vacuum distillation at 200–230 °C.^5^ Alternative sources, such as plant-derived SQE from vegetable oils and refining byproducts, have attracted attention due to their sustainability and potential to meet market demand.^6^ Olive oil, with ∼564 mg/100 g of SQE, is the main plant source for SQE extraction.^4^

Recent advancements in synthetic biology have enabled the engineering of microorganisms for enhanced SQE production, offering the potential for large-scale manufacturing.^7^ Additionally, phytosqualene can be derived from trans-β-farnesene, which is produced through the fermentation of sugar cane by genetically modified *Saccharomyces cerevisiae* and then converted to SQA via catalytic dimerization and hydrogenation.^8^

The recovery of SQE from olive pomace (OP) oil has emerged as a viable alternative, particularly when using deodorizer distillates (DDs) obtained during the physical refining of the oil. OP contains approximately 10% oil by dry weight and is typically extracted using hexane. In a previous study, we have demonstrated that 2-methyltetrahydrofuran (2-MeTHF) is a valuable green alternative to hexane for the extraction of OP oil.^9^ The main advantages of this solvent are its biodegradability, its fully biobased character and its toxicological profile, which is safer than that of hexane. In addition, its use in industrial systems is economically advantageous compared to other alternatives such as ethanol (EtOH), due to its lower latent heat of vaporization (364 vs 846 kJ/kg) and supercritical CO_2_, which requires higher capital investment.^10^ 2-MeTHF has also already been approved for food production in Europe.^11^

Due to its high acidity, OP oil requires refining and deodorization. OP-DDs have proven to be a rich source of SQE.^6^ Bondioli et al., have analyzed various types of OP-DDs and have reported SQE yields ranging from 224 to 452 mg/g DDs.^12^ Two processes are typically used to increase SQE concentration; the esterification of fatty acids and separation of high purity (>95%) SQE via vacuum distillation, and the extraction of the unsaponifiable fraction with hexane to recover SQE with a maximum purity of around 80%.^5^

Due to its chemical structure, in particular its high degree of unsaturation, SQE is very unstable and easily oxidized.^13^ One of the key transformations of SQE is its catalytic hydrogenation to squalane (SQA).^14^ SQA is widely used in nutraceutical and cosmetics ingredients, because of its proven emollient and moisturizing properties.^13^ Regardless of its source, SQE must be converted to SQA at over 92% purity and with an iodine value of below 1.00 (preferably <0.10) for cosmetic use.^5^

SQE hydrogenation is traditionally conducted in stirred tank reactors, with high loadings of Ni- and Pd-based catalysts under harsh conditions and/or over extended reaction times.^1^ Suboptimal process conditions are typically employed on an industrial scale in a two-step, one-pot process. The first step is carried out at low H_2_ pressure, followed by a second step at higher H_2_ pressure and higher temperature to achieve complete reduction. Ni-based catalysts have long been used in hydrogenation processes, mainly due to Ni’s lower cost compared to Pd.^15^ However, Ni-catalysts require higher metal loading and temperatures. Recently, various Pd-based catalysts have been used for the hydrogenation of SQE, either with or without solvent, including carbon-nanotube-supported Pd,^1^ Pd/C,^16^ sol–gel-entrapped SiliaCat Pd(0),^14,17^ and Pd/clay.^18^

Current hydrogenation processes make up about 40% of the total production cost of SQA. To reduce these costs, there is a need for new hydrogenation processes that operate at lower pressures, temperatures and at shorter reaction times. Previous studies have demonstrated that the use of solvents, such as EtOH and isopropanol, during the catalytic hydrogenation of SQE enables the reaction to proceed under significantly milder conditions than under neat reactions, resulting in improved yields and higher overall process efficiency.^14,16^ Compared to EtOH and isopropanol, 2-MeTHF offers two key advantages: first, its dual function in SQE extraction from OP and hydrogenation eliminates the need for solvent exchange, which can simplify process integration within the same production site; second, its significantly lower latent heat of vaporization reduces energy consumption during solvent distillation from the final product, which lowers overall process costs. For this reason, the use of 2-MeTHF as a green solvent has been investigated.

To the best of our knowledge, this is the first study to experimentally demonstrate an integrated route for SQE extraction from OP and its hydrogenation in 2-MeTHF without solvent exchange. The objective is to assess the efficiency of SQE recovery from OP using 2-MeTHF and to optimize its catalytic hydrogenation to SQA under mild conditions. Particular attention is given to identifying optimal reaction conditions, including temperature, H_2_ pressure and catalyst loading, to maximize SQA yield. The study also investigates catalyst reusability over multiple cycles. The dual functionality of 2-MeTHF as both extraction and hydrogenation solvent (Figure 1) supports process integration and sustainability, offering a greener and more streamlined approach to SQA production.

**Figure 1:**
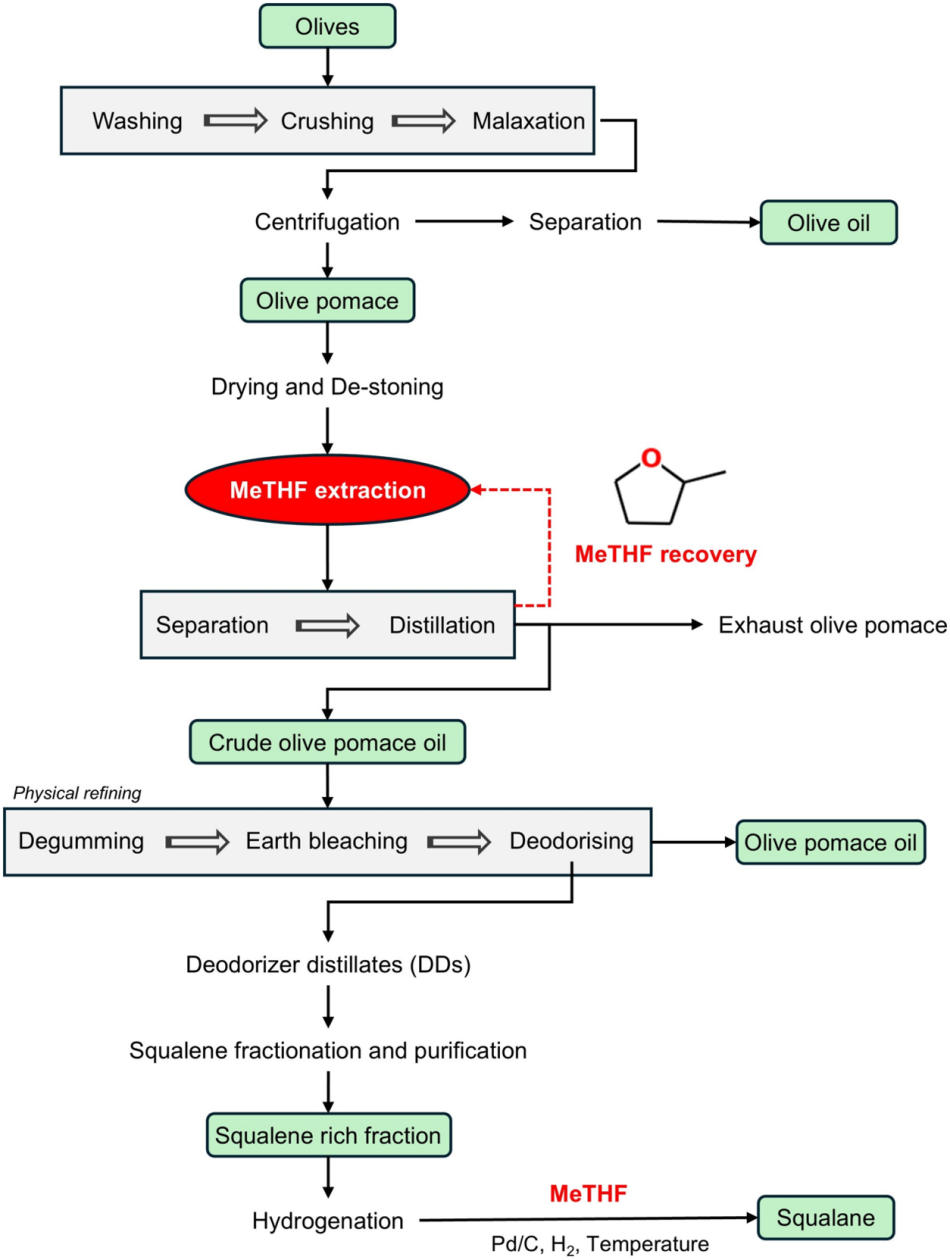
Integrated industrial process for SQA production from OP using 2-MeTHF as a green solvent.

## 2 Results and Discussion

### 2.1 OP Oil Extraction with 2-MeTHF

The use of new green solvents and technologies for the extraction of oil and bioactive compounds from OP has been the subject of much research in recent years.^19,20^ Traditionally, hexane has been the solvent of choice for vegetable-oil extraction, including OP oil, due to its technical proprieties that allow for cost-effective extraction processes and high oil yields. However, hexane is highly toxic, flammable and environmentally harmful,^21^ prompting the EFSA to highlight the need for a re-evaluation of its safety as an extraction solvent in food production.^22^ For this reason, the development of new, safe extraction processes is essential.

In this study, a significantly higher (*p* < 0.05) OP oil yield was obtained with 2-MeTHF than with hexane (Figure 2). This is consistent with previous findings, which emphasizes its greater ability to extract nontriacylglyceride compounds, including phospholipids^23^ and polyphenols.^9^ Some studies have already investigated the extraction of other vegetable oils, on both the laboratory,^23^ and semi-industrial scales^24^ using 2-MeTHF. Moreover, 2-MeTHF can be implemented on an industrial scale using existing hexane equipment, requiring only minor modifications.^10^

**Figure 2:**
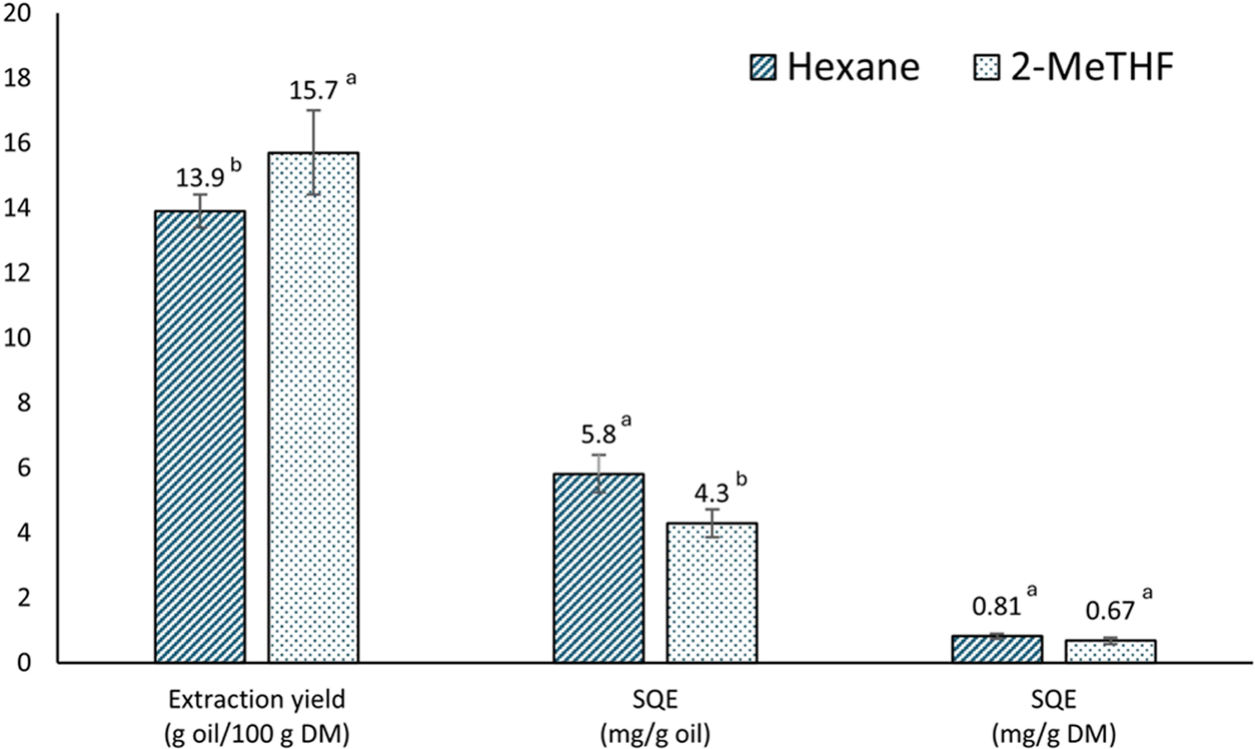
Extraction yield and SQE content in OP oil obtained with hexane and 2-MeTHF. Different letters indicate significant differences (*p* < 0.05).

Initial SQE concentration in crude OP oil plays a critical role in determining the amount of SQE recovered during oil refining. The SQE content in OP oil is generally reported to be between 0.5 and 6 mg/g oil.^25^ In this study, the SQE concentration in the oil extracted using hexane (5.8 mg/g oil) was higher than that obtained using 2-MeTHF (4.3 mg/g oil). This difference is mainly due to the coextraction of additional polar compounds by 2-MeTHF, which decreases the relative SQE content in the oil. This effect was demonstrated in studies on OP, where 2-MeTHF extracted more polar compounds, including polyphenols, than hexane.^9^ Similarly, Claux et al. (2021) have reported that 2-MeTHF improves the solubilization of phospholipids, recovering more than twice the amount obtained with hexane from soybean flakes.^23^ Nevertheless, due to the higher extraction yield of 2-MeTHF, approximately 83% of the total SQE in the OP was recovered, considering hexane extraction as the benchmark (100%).

During the physical refining of OP oil, a significant fraction of SQE is transferred to the DDs, where it is concentrated. Therefore, a higher SQE content in crude OP oil directly translates into higher SQE recovery in DDs. Overall, these findings highlight the potential of 2-MeTHF as an environmentally friendly solvent for OP oil extraction and SQE recovery. In addition, 2-MeTHF can also serve as a solvent for the hydrogenation of SQE from OP-DDs in an integrated process from SQE extraction to SQA production. The following sections deal with the optimization of SQE hydrogenation in 2-MeTHF as solvent.

### 2.2 SQE Hydrogenation: Conditions Optimization

Previous studies have shown that although the reaction can proceed in solvent-free conditions (neat), the presence of solvents (e.g., EtOH and isopropanol) ensures more efficient H_2_ transfer, leading to faster conversion and better selectivity in SQE hydrogenation to SQA.^16^ In solvent-free reactions, much harsher conditions, in terms of temperature, H_2_ pressure and time, are required to achieve high yields. These results can be explained by the significantly lower solubility of H_2_ in SQE compared to SQE/EtOH or isopropanol mixtures.^14^

In this study, 2-MeTHF was compared with EtOH as a solvent for the microwave (MW)-assisted hydrogenation of SQE (Figure 3). The reaction conditions, including temperature, H_2_ pressure and reaction time were optimized to maximize conversion and selectivity, using a commercial SQE sample (≥98 wt %).

**Figure 3:**
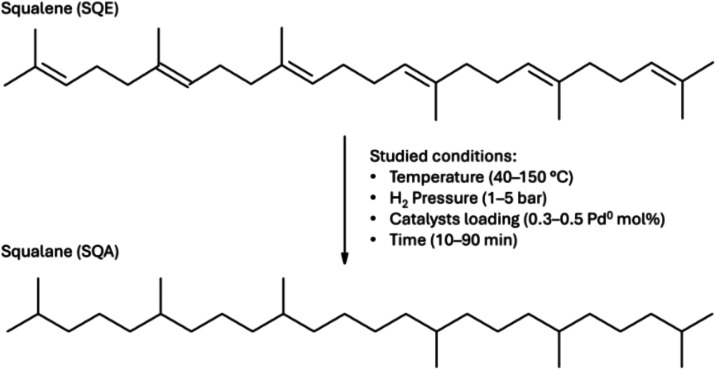
SQE hydrogenation to SQA in 2-MeTHF.

First, the effect of reaction temperature was evaluated, while keeping the catalyst loading (0.5 mol % Pd/C catalyst), H_2_ pressure (5 bar) and reaction time (1 h) constant. The results are presented in Table 1, where the results under neat conditions and with EtOH as the solvent are also presented for comparison.

**Table 1:** Influence of Temperature on SQE Conversion and SQA Yield^a^

solvent	temperature (°C)	SQA yield (%)	SQE conversion (%)
2-MeTHF	150	81.1	100
	100	85.4	100
	80	87.5	100
	60	100	100
	40	43.0	100
EtOH	100	100	100
	80	100	100
	60	100	100
	40	69.7	100
neat	150	100	100
	100	84.2	100
	80	30.3	100

a Reaction conditions: 0.5 mol % Pd^0^ catalyst, 5 bar H_2_, 1 h.

The presence of a solvent dramatically improved the hydrogenation process at lower temperatures. Both EtOH and 2-MeTHF facilitate the reaction but show significant differences in performance at different temperatures. With EtOH, complete SQE conversion to SQA is achieved over a wide temperature range (60–100 °C). This confirms that EtOH effectively improves the diffusion of H_2_ within the reaction medium, resulting in a more efficient hydrogenation process.

2-MeTHF shows optimal performance at 60 °C, achieving complete SQA conversion. However, at higher temperatures (80, 100, and 150 °C), the SQA yield decreases despite the complete conversion of SQE. The higher volatility of 2-MeTHF compared to EtOH may lead to greater solvent evaporation at elevated temperatures, which can reduce H_2_ solubility in the reaction medium. GC-MS analysis confirmed the presence of partially hydrogenated intermediate products (MW: 412, 414, 416, 418, and 420) near the retention time of SQA when conversion was incomplete (see Supporting Information Figure S1 and S2). At 40 °C, SQA yield decreased when using the solvents, with the reaction in EtOH being more effective than in 2-MeTHF (70% vs 43% SQA yield). Nevertheless, the lower latent heat of vaporization of 2-MeTHF compared to EtOH and isopropanol results in reduced energy demand during solvent recovery by distillation, thus contributing to lower overall process costs. A comparison of boiling points and latent heat values is presented in Figure S3.

Under neat conditions, complete conversion is still achieved, but the SQA yield is significantly lower, especially at temperatures below 100 °C. These results suggest that the absence of a solvent negatively impacts upon both the kinetics and selectivity of the reaction, meaning that higher reaction temperatures are required to achieve complete conversion because of mass-transfer limitations. In a previous study, Pandarus et al., achieved 99% conversion to SQA at 70 °C under solvent-free conditions using 3 bar H_2_, 0.2 mol % SiliaCat Pd(0) with high-purity SQE (98 wt %).^17^ However, when the temperature was lowered to 50 °C, the SQA yield dropped significantly to only 45%. Compared to our study, these reactions required a longer reaction time (24 h vs 1h). In addition, much higher temperatures were required for lower purity SQE (82 wt %) to achieve comparable conversion and selectivity, showing how impurities can severely impact the efficiency of the process.

Moving now to the effect of other reaction parameters, both increased H_2_ pressure and higher catalyst loading have a positive effect on reaction yield, as shown in Table 2. Higher pressure improves H_2_ availability, while higher catalyst loading increases the number of active sites.

**Table 2:** Influence of H_2_ Pressure and Catalyst Loading on SQE Conversion and SQA Yield in 2-MeTHF^a^

entries	Pd^0^ (mol %)	H_2_ bar	SQA yield %	SQE conversion %
1	0.3	1	22.6	100
2	0.5	1	48.7	100
3	0.3	3	57.0	100
4	0.5	3	96.3	100
5	0.3	5	82.3	100
6	0.5	5	100	100

a Reaction conditions: 60 °C, 1 h.

As the H_2_ pressure increased from 1 to 5 bar, a notable improvement in SQA yield was observed for all catalyst loadings. Moreover, increasing the catalyst loading from 0.3 mol % to 0.5 mol % consistently improves SQA yield for all H_2_ pressures tested. At 1 bar, SQA yield almost doubled when the catalyst loading was increased from 0.3 mol % to 0.5 mol % (entries 1–2). A similar result was observed at 3 bar (entries 3–4) and 5 bar H_2_ (entries 5–6).

The relevance of the reaction parameters in the solvent-free hydrogenation of SQE to SQA follows the order: Pd loading > temperature > pressure.^17^ Indeed, under neat conditions, complete conversion to SQA was achieved only at catalyst concentrations of 0.5 mol %.

In this study, the optimal conditions identified include a H_2_ pressure of 3 bar, a temperature of 60 °C, and a catalyst loading of 0.5 mol %. By contrast, neat industrial hydrogenation processes often rely on more demanding conditions. For instance, the Ni-Kieselguhr catalyst (0.05 wt %) operates at 200 °C under H_2_ pressures ranging from 4 to 30 bar in a two-step process lasting 6–7 h.^5^ Impure SQE substrates, such as those derived from OP-DDs, require even harsher conditions and additional purification steps.^14^ Another catalyst commonly employed on an industrial scale is Pd/C (loading of 0.25 mmol g^–1^ Pd). The reaction is typically carried out at a temperature of 150–160 °C, initially under a H_2_ pressure of 3 bar, which is subsequently increased to 70 bar.^26^

Reaction kinetics were then evaluated, as illustrated in Figure 4. These results reveal a rapid increase in SQA yield over the first 50 min, reaching approximately 89%. The yield of SQA then continues to rise, approaching a near plateau, at around 96%, after 60 min. Considering these findings, a reaction time of 1 h was selected as being optimal.

**Figure 4:**
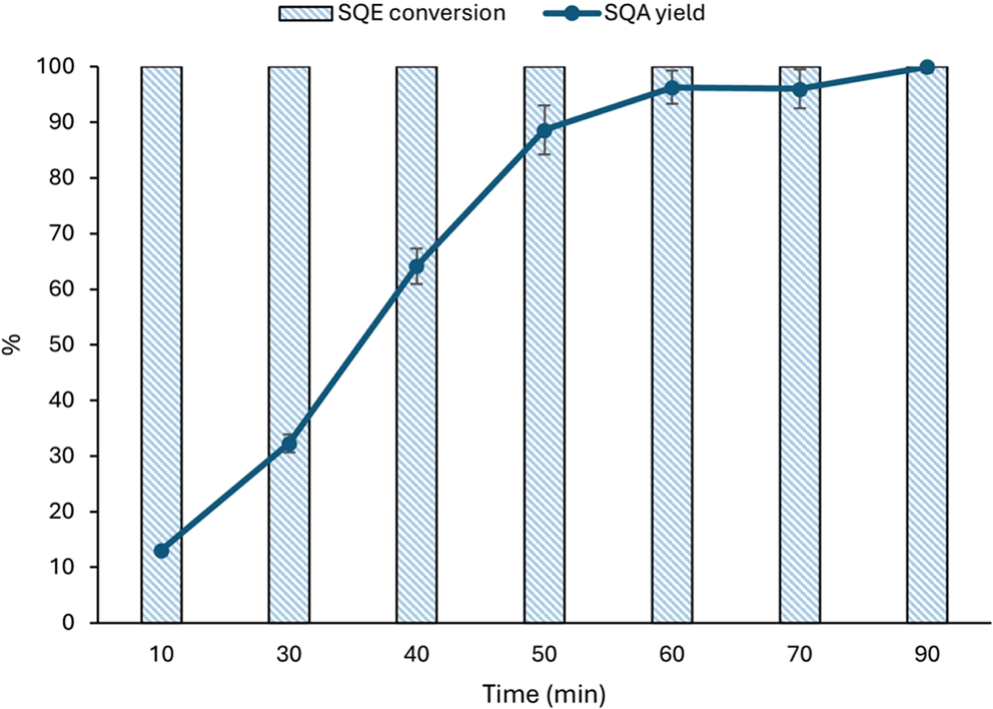
SQE hydrogenation kinetics using 2-MeTHF (0.5 mol % Pd^0^, 60 °C, 3 bar H_2_).

### 2.3 Catalyst Reusability Tests

The recyclability of the catalyst in heterogeneous SQE hydrogenation was investigated under the optimized conditions in 2-MeTHF as the solvent (0.5 mol % Pd/C, 60 °C, 3 bar, 1h). The catalyst was collected by centrifugation after each run, washed twice with methanol, dried at room temperature and reused in a subsequent run.

Figure 5 shows data related to SQE hydrogenation across the five cycles of catalyst recycling. It is evident that while the conversion of SQE was always complete, the yield of SQA progressively decreases from 96% in the first cycle to 22% in the fifth run.

**Figure 5:**
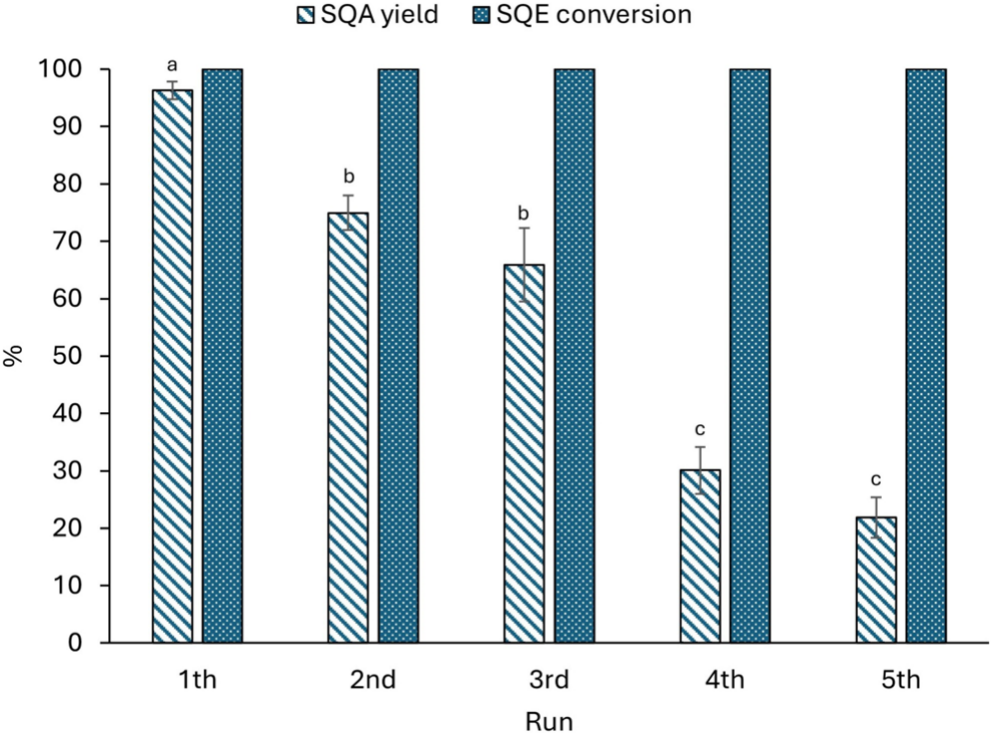
Reusability of the Pd/C catalyst in SQE hydrogenation in 2-MeTHF. Different letters indicate significant differences (*p* < 0.05).

The leaching of Pd^0^ from the Pd/C catalyst into the reaction mixture is a potential cause of reduced efficiency.^27^ Over the recycling tests, the loss of active Pd^0^ could lead to a decrease in catalytic activity, thereby significantly reducing (*p* < 0.05) SQA yield. In this study, after the fifth run, the catalyst underwent a 33.02% Pd^0^ loss, decreasing from 107.7 (fresh catalyst) to 72.6 g Pd^0^/kg in the spent catalyst, leading to a progressive decrease in SQA yield across reaction cycles.

The leaching of Pd and Si from a SiliaCat Pd^0^ catalyst during SQE hydrogenation has been investigated by Pandarus et al.^14^ Various solvents were tested, including EtOH, methanol, 2-MeTHF and isopropanol. Across all conditions, leached Pd and Si levels in the crude product remained below 5 and 10 ppm, respectively, although 2-MeTHF showed slightly higher values. Leaching raises operational costs due to the need for more frequent catalyst regeneration. Additionally, catalyst leaching reduces process efficiency, resulting in lower yields and prolonged reaction times. In this context, SiliaCat Pd(0) may offer a more sustainable alternative, enabling complete hydrogenation under mild conditions with minimal metal leaching and improved recyclability. Overall, the economic feasibility of using 2-MeTHF in large-scale operations, particularly with catalyst recycling, requires further evaluation.

### 2.4 SQA from OP-DDs

The extraction of SQE from OP oil has become a viable alternative to shark-derived SQE, especially when using OP-DDs recovered during the physical refining of oil. This byproduct is a complex mixture containing free fatty acids, phytosterols, tocopherols, sterol esters, hydrocarbons and the breakdown products of fatty acids, aldehydes, ketones and acylglycerols.^28^

The characteristics of the OP-DDs used in this study are summarized in Table 3. The OP-DDs appeared as a yellowish semisolid with a low moisture content (0.46 wt %). Approximately 20% of the OP-DDs was made up of an unsaponifiable fraction, which included nonfatty-acid compounds such as SQE and phytosterols. SQE constituted 6.85 wt % of the total OP-DDs, thus it made up 34.8 wt % of the unsaponifiable fraction. However, SQE content was lower here than the previously reported concentrations of 22–45 wt % SQE in OP-DDs^12^ and around 60–75 wt % of the unsaponifiable fraction.^28^

**Table 3:** Chemical Analysis of OP-DDs

	OP-DDs
physical appearance	yellowish semisolid
moisture (wt %)	0.46 ± 0.06
unsaponifiable (wt %)	19.68 ± 0.79
SQE (wt %)	6.85 ± 0.10
SQE (wt % of OP-DDs unsaponifiable)	34.79 ± 0.51

Given the low SQE concentration, two purification methods were evaluated; saponification and molecular distillation, the latter of which was performed after the methyl esterification of fatty acids in OP-DDs. Both approaches led to an approximately 5-fold increase in SQE concentration. However, the low initial concentrations resulted in a final SQE content of 34.8 wt % in the unsaponifiable fraction and 34.3 wt % in the nonvolatile fraction being recovered after molecular distillation. Both purified fractions were tested under the optimized hydrogenation conditions, but no SQA formation was observed due to low SQE purity. To improve this, flash chromatography was applied to the unsaponifiable fraction, which showed slightly higher SQE content than the nonvolatile residue from molecular distillation. This step was necessary due to the unusually low SQE concentration in the OP-DD sample. However, flash chromatography has limited scalability, and under typical SQE levels reported in the literature, extraction of the unsaponifiable fraction alone is generally sufficient to achieve purities up to 80%.^5^ This additional purification step yielded an SQE purity of 59.1 wt % with an overall recovery rate of 85.1%.

This purified product was tested under optimized hydrogenation conditions (2-MeTHF solvent, 0.5 mol % Pd catalyst, 60 °C, 3 bar H_2_, 1 h), but residual impurities negatively affected the reaction, resulting in no detectable SQA. To address this, a second test was performed with an increased catalyst concentration (1 mol % Pd) and higher H_2_ pressure (5 bar), leading to complete SQE conversion and a SQA yield of 19.8%. Overall, these findings underscore the critical impact of product impurities, likely due to catalyst poisoning, on reaction efficiency. Pandarus et al., (2015) have shown that the purity of SQE strongly influences hydrogenation efficiency. While 98 and 92 wt % pure samples were completely converted to SQA within 4 and 8 h, respectively, an 82 wt % sample was only partially hydrogenated, with no further progress after 24 h.^14^

These results underline the need for either higher SQE purity in the starting material or the implementation of additional purification steps to achieve efficient hydrogenation and maximize SQA yield.

## 3 Conclusions and Future Perspectives

This study presents an integrated approach for the valorization of OP through SQE extraction and hydrogenation using a single green solvent, 2-MeTHF, and highlights its potential for sustainable SQA production while addressing the environmental and safety concerns associated with hexane extraction methods.

Using hexane as a benchmark, SQE extraction with 2-MeTHF achieved approximately 83% recovery. The higher coextraction of polar compounds by 2-MeTHF reduced the SQE concentration in the crude OP oil. Further investigation is needed to assess how this could affect SQE concentration in OP-DDs after refining.

SQE hydrogenation in 2-MeTHF demonstrated high efficiency under milder conditions (60 °C, 3 bar H_2_, 0.5 mol % Pd/C), achieving complete conversion within one hour. This represents a significant improvement over conventional industrial hydrogenation, which requires harsher conditions (e.g., 200 °C, 4–30 bar H_2_) and extended reaction times (6–7 h). Additionally, compared to other solvents (e.g., EtOH and isopropanol), the lower distillation energy demand of 2-MeTHF may significantly reduce overall process costs. However, catalyst deactivation due to leaching remains a key challenge during catalyst recycling.

In order to evaluate industrial applicability, SQE was purified from industrial OP-DDs via saponification and molecular distillation, achieving a purity of ∼34 wt %. Flash chromatography further increased the purity to 59.1 wt % with a recovery rate of 85.1%. However, residual impurities, which were probably due to catalyst poisoning, hindered hydrogenation. While increases in catalyst loading and H_2_ pressure (1 mol % Pd/C, 5 bar) enabled complete SQE conversion, the SQA yield remained low (19.8%). Future studies should focus on optimizing SQE purification from OP-DDs and improving catalyst recyclability (e.g., exploring alternative catalysts) to strengthen the industrial potential of this process.

## 4 Materials and Methods

An overview of the experimental approaches is illustrated in Figure 6.

**Figure 6:**
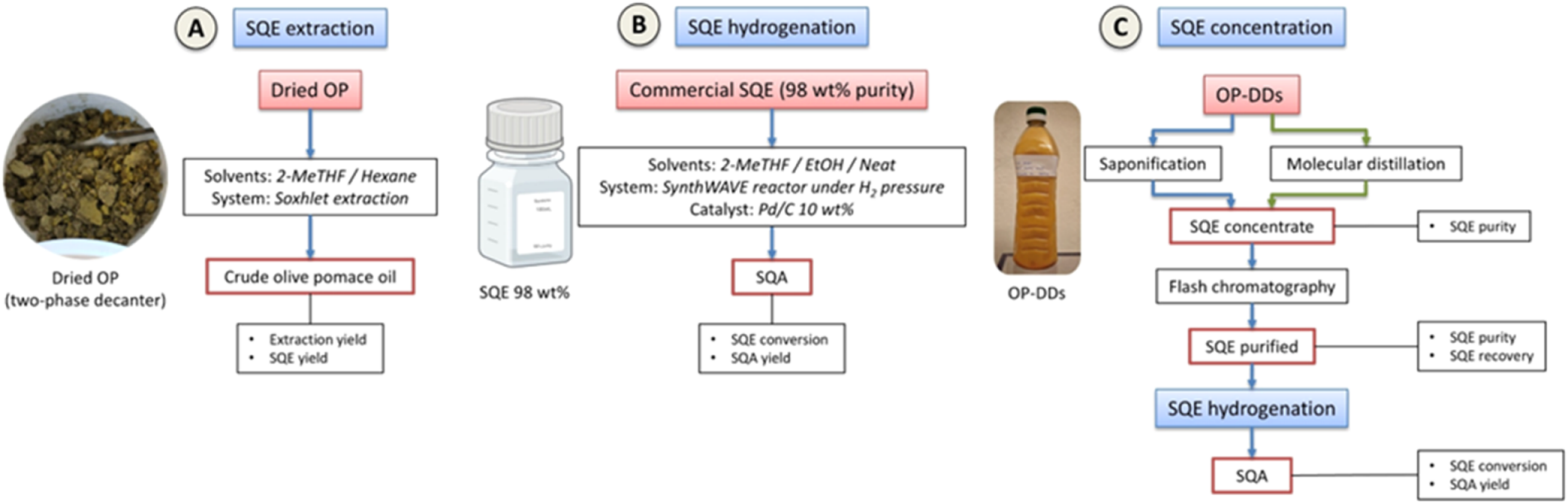
Overview of the experimental approaches for SQE extraction, hydrogenation and purification.

### 4.1 Reagents and Instrumentation

Solvents (EtOH ≥ 99 wt %, *n*-hexane ≥ 97 wt %, 2-methyltetrahydrofuran ≥ 99 wt %), reagents (chloroform ≥ 99 wt %, methanol ≥ 99.9 wt %, p-toluenesulfonic acid ≥ 98.5 wt %), the catalyst (Pd/C 10 wt %), and the products (SQE ≥ 98 wt %, SQA ≥ 98 wt %) were purchased from Merck (Darmstadt, Germany). OP-DDs were supplied by Altec srl.

Chromatographic separation was conducted using a CombiFlash rf 200 system by Teledyne ISCO with RediSep reverse-phase C-18 silica columns. The hydrogenation step was performed using a SynthWAVE reactor (Milestone Srl, Bergamo, IT).

The molecular distillation of DDs was performed using a KDL-4 short-path distillation system (Legallais, France) equipped with a thermostatic feed, an evaporation surface of 0.04m^2^ with 4 roller wipers and an internal condenser, a cold trap and a vacuum pump.

GC-MS analyses were performed on an Agilent 6850 system equipped with an Agilent 5973 quadrupole detector and a MEGA-5 MS low-polarity column.

### 4.2 Oil Solvent Extraction

OP was sourced from Moulin Castelas (Baux-de-Provence, France), which operates a two-phase olive oil extraction system. The proximate composition of the dried OP was reported in our previous study: ash, 4.2%; oil, 13.7%; protein, 6.6%; total phenolic content, 2.2%; and fibers, 73.28%.^9^ Dried OP was extracted using hexane and 2-MeTHF with Soxhlet apparatus over 10 cycles, see Figure S4A. Solvents were evaporated using a rotavapor at 40 °C under a vacuum pressure of 30 mbar. The resulting crude extracts were centrifuged at 8875 G for 5 min to remove any solid particles. The extraction yield was determined based on the mass of oil extract relative to the extracted OP. The SQE content in OP oil was quantified using GC-MS analysis. All experiments were performed in triplicate.

### 4.3 Hydrogenation Process

Experimental conditions were optimized using commercial SQE (98 wt % purity), see Figure 6. The hydrogenation of SQE was performed in a SynthWAVE reactor (Milestone Srl, Bergamo, IT) under H_2_ pressure, using Pd/C 10 wt % as the catalyst (see Figure S4B). A similar experimental setup was used by Bucciol et al., in a previous study.^16^ The SQE (1 mmol) and catalyst (0.3–0.5 Pd^0^ mol % of SQE) were directly weighed in a 20 mL glass vial. The reaction temperature was continuously monitored using an internal probe, and variations were maintained within ± 1 °C of the set temperature. The system was pressurized with H_2_ before microwave activation, while magnetic agitation ensured homogeneous mixing throughout the reaction. During the reactions conducted in solvent, 2 mL of either anhydrous EtOH or 2-MeTHF were added. At the end of the process, the system was cooled to room temperature and the pressure was released. The reaction mixture was then centrifuged at 4200 rpm for 5 min to precipitate the catalyst. The SQE conversion and SQA yield were assessed by GC–MS.^14,16^ Calibration curves were performed for SQE and SQA (0.5–10 mg/mL, R2 ≥ 0.999) using standard solutions. All experiments were performed in duplicate.

### 4.4 Catalyst Reusability Tests

The efficiency of the catalyst over several successive runs was investigated, and five consecutive runs were conducted in total. After each reaction, the heterogeneous catalyst was separated from the reaction mixture by centrifugation, washed twice with methanol and then dried. After the fifth run, the Pd content was analyzed using ICP-OES with a PerkinElmer Optima 7000 (PerkinElmer, Norwalk, CT, USA) spectrometer.

### 4.5 Saponification and Esterification of OP-DDs

The saponification of OP-DDs was performed with a solution of KOH in EtOH (2N) at 80 °C for 60 min.^29^ After cooling to room temperature, the mixture was transferred to a separating funnel and extracted with *n*-hexane many times (at least six) until the organic layer appeared clear. The solvent was then evaporated to recover the unsaponifiable matter. For esterification, 30 g of OP-DD, methanol (50 mL) and concentrated sulfuric acid (1% of OP-DD) were placed in a 500 mL round-bottom flask. The mixture was stirred magnetically at 80 °C for 2 h. The mixture was allowed to cool to room temperature and washed with water to remove any residual acid. The excess methanol was removed by evaporation under reduced pressure. The resulting unsaponifiables and esterified products were then analyzed using GC-MS.

### 4.6 SQE Purification

In this study, SQE purification from unsaponifiable OP-DDs was achieved using flash chromatography in a C-18 silica column. The chromatographic method used gradient elution with solvents A (water), B (isopropanol) and C (methanol), starting at 2% A, 0% B and 98% C, followed by 2% A, 80% B and 8% C at 16 min, and 0% A, 100% B, and 0% C from 18 to 22 min. The purification process was monitored by UV–vis absorbance at 254 and 214 nm. Collected fractions were concentrated via evaporation under vacuum and subsequently analyzed by GC-MS. The methyl-esterified OP-DD sample was distilled using a short-path distillation system (see Figure S4C) under the following conditions: 140 °C at 0.5 mbar pressure, with a feed flow rate of 3 mL/min, a feed temperature of 40 °C, a condenser temperature of 20 °C, and a wiper rotational speed of 400 rpm. Molecular distillation of 46.3 g of methyl esterified OP-DDs yielded two distinct fractions: a volatile distillate (rich in fatty acid methyl esters) weighing 36.7 g, and a nonvolatile residue (enriched in SQE) weighing 5.0 g. Approximately 4.6 g of material was unaccounted for, likely due to handling losses and retention within the apparatus. Based on its SQE concentration, the recovery in the nonvolatile fraction was estimated at approximately 55%. Both fractions were collected and stored at – 18 °C until further analysis.

### 4.7 Statistical Analysis

Data were analyzed using one-way ANOVA. Where appropriate, multiple comparisons of means were performed using Tukey’s HSD test at a significance level of 5%.

## Supplementary Material



## Notes

GC-MS chromatograms of hydrogenation products under different reaction conditions and MS spectra of partially hydrogenated compounds are reported (PDF)

## #

URD Agro-Biotechnologies Industrielles (ABI), CEBB, AgroParisTech, 51110 Pomacle, France
