# Supplementary material for: From Olive Pomace to Squalane: A Green Chemistry Route Using 2‑Methyltetrahydrofuran
Source: ACS Omega. 2025 Jul 25;10(30):33426–34. doi: 10.1021/acsomega.5c03685 (PMC12332605; doi:10.1021/acsomega.5c03685)

From Olive Pomace to Squalane: A Green Chemistry Route Using 2-Methyltetrahydrofuran

Christian Cravotto [a,b], Fabio Bucciol [a], Jean-Baptiste Mazzitelli [b], Emmanuel Petitcolas [b], Anne-Sylvie Fabiano-Tixier [b], and Silvia Tabasso [a].*

[a] Department of Drug Science and Technology, University of Turin, Via P. Giuria 9, 10125 Turin, Italy; fabio.bucciol@unito.it (F.B.).

[b] Avignon Université, INRAE, UMR SQPOV, F-84000 Avignon, France.

Figure S1. GC-MS chromatogram of hydrogenated products under different reaction conditions: (A) Complete hydrogenation at 60 °C for 1 h, (B) Partial hydrogenation at 40 °C for 1 h.

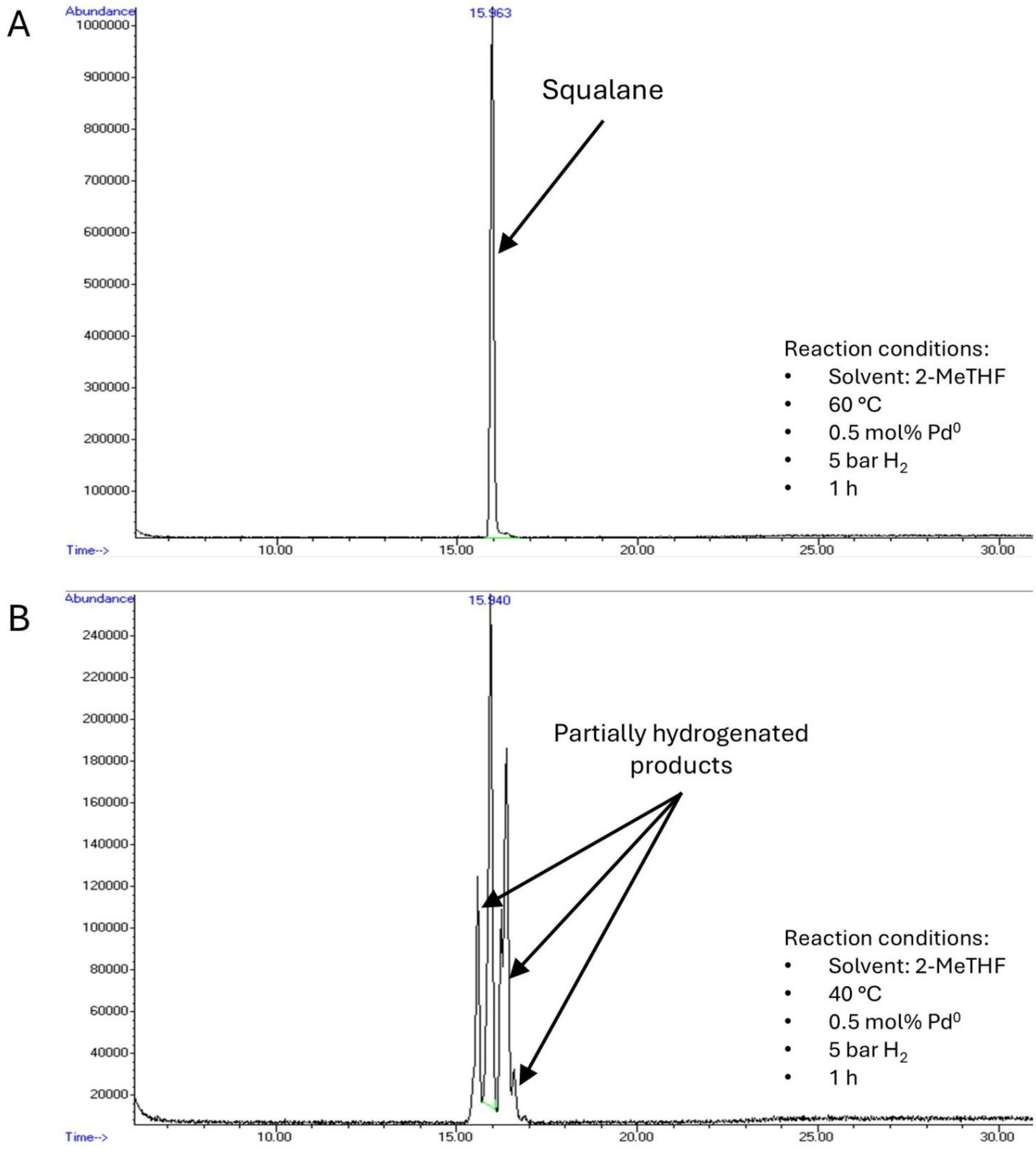

Figure S2. MS spectra of the partially hydrogenated products: (A) peak with RT = 16.229; (B) peak with RT = 16.391.

A

B

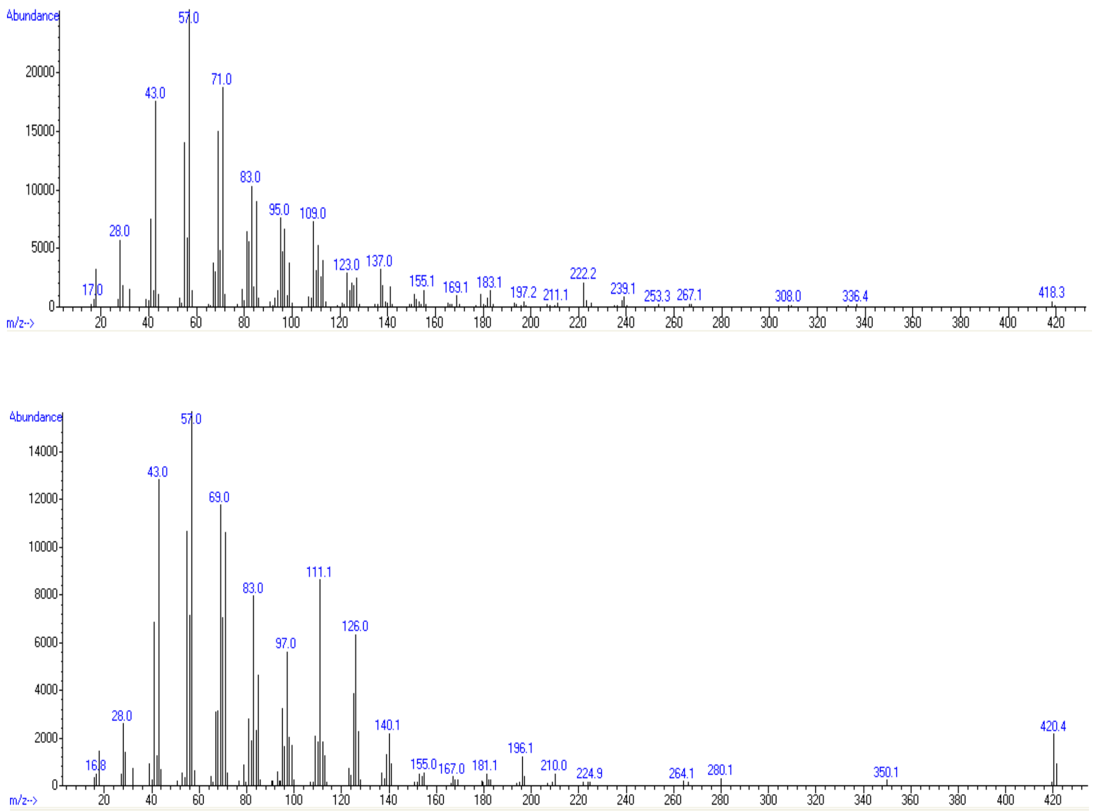

Figure S3. Latent heat of vaporisation and boiling point of selected solvents used in SQE extraction and hydrogenation.

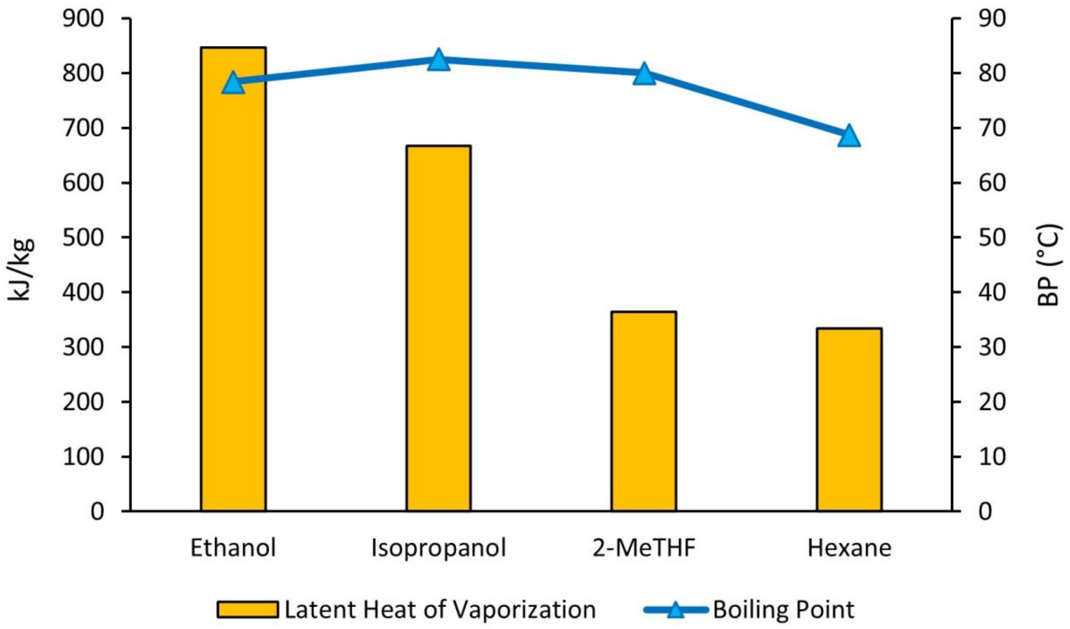

Figure S4. Experimental setups used over the study: Soxhlet extraction of OP (A), catalytic hydrogenation in the SynthWAVE reactor (B), and short-path distillation for SQE purification (C).

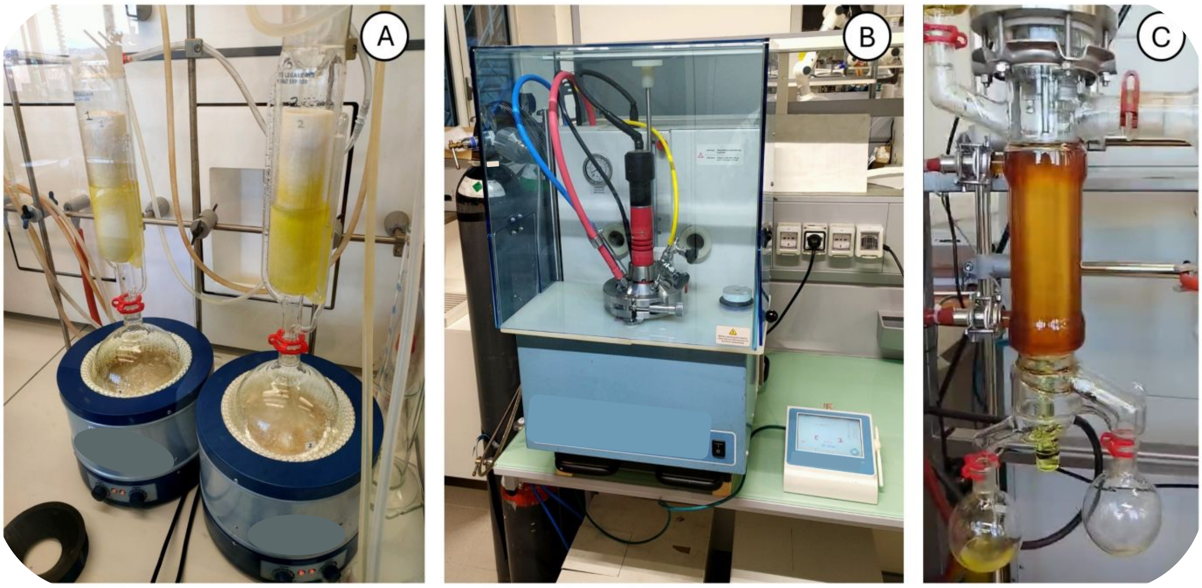

Supplement: Supplementary file 1 [file ao5c03685_si_001.pdf]
